# Interrelation of Natural Polyphenol and Fibrosis in Diabetic Nephropathy

**DOI:** 10.3390/molecules30010020

**Published:** 2024-12-25

**Authors:** Ye Ma, Jiakun Wang, Juyue Fan, Huiyang Jia, Jinyao Li

**Affiliations:** 1 School of Pharmaceutical Sciences and Institute of Materia Medica, Xinjiang University, Urumqi 830017, China; maye@xju.edu.cn (Y.M.); 107552203670@stu.xju.edu.cn (J.W.); fanjuyue@stu.xju.edu.cn (J.F.);; 2 Xinjiang Key Laboratory of Biological Resources and Genetic Engineering, College of Life Science and Technology, Xinjiang University, Urumqi 830017, China

**Keywords:** diabetic nephropathy, polyphenol, fibrotic niche, oxidative stress, inflammation

## Abstract

Diabetic nephropathy (DN) is a common and serious complication of diabetes mellitus and a major cause of end-stage renal disease (ESRD). Renal fibrosis, which corresponds to excessive deposition of extracellular matrix and leads to scarring, is a characteristic feature of the various progressive stages of DN. It can trigger various pathological processes leading to the activation of autophagy, inflammatory responses and a vicious circle of oxidative stress and inflammation. Although it is known that DN can be alleviated by mechanisms linked to antioxidants, reducing inflammation and improving autophagy, how to improve DN by reducing fibrosis using natural polyphenols needs to be studied further. Nowadays, natural polyphenolic compounds with excellent safety and efficacy are playing an increasingly important role in drug discovery. Therefore, this review reveals the multiple mechanisms associated with fibrosis in DN, as well as the different signaling pathways (including TGF-β/SMAD, mTORC1/p70S6K, JAK/STAT/SOCS and Wnt/β-catenin) and the potential role in the fibrotic niche. In parallel, we summarize the types of polyphenolic compounds and their pharmacodynamic effects, and finally evaluate the use of polyphenols to modulate relevant targets and pathways, providing potential research directions for polyphenols to improve DN. In summary, the problem of long-term monotherapy resistance can be reduced with natural polyphenols, while reducing the incidence of toxic side effects. In addition, potential targets and their inhibitors can be identified through these pathways, offering potential avenues of research for natural polyphenols in the pharmacological treatment of multisite fibrosis.

## 1. Introduction

Diabetes mellitus and its complications have become one of the major global public health challenges, with approximately 40% of diabetic patients eventually developing diabetic nephropathy (DN) [1]. DN is the most common microvascular complication among diabetic patients and the cause of end-stage kidney disease (ESRD) [2], and its pathogenesis involves multiple metabolic disorders. Under hyperglycemic conditions, metabolic byproducts such as advanced glycation end-products (AGEs) and oxidative stress damage the kidney structures and function, resulting in a decline in glomerular filtration function [3]. Moreover, DN not only affects kidney function but is also an independent risk factor for cardiovascular events [4]. At present, DN is mainly treated by dialysis, kidney transplantation and gene therapy, which consume significant medical resources and impose a considerable economic and psychological burden on patients. Therefore, any improvement in the treatment of DN has important clinical significance [5].

**Figure 1:**
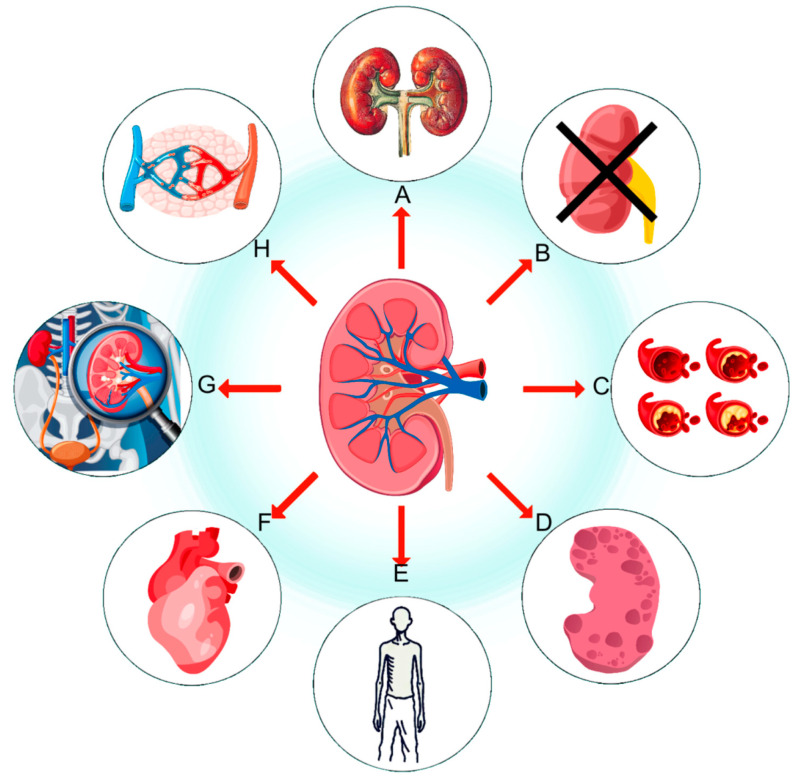
Major manifestations and complications of diabetic nephropathy. (**A**) Kidney fibrosis, (**B**) impaired kidney function, (**C**) elevated blood glucose and lipids, (**D**) kidney structural damage, (**E**) weight loss, (**F**) cardiovascular disease, (**G**) uremia, and (**H**) edema.

The pathogenesis of DN is highly complex, involving various metabolic and hemodynamic alterations [6]. Among these, kidney fibrosis is considered a critical factor in the development of diabetic nephropathy [7]. Research indicates that the progression of fibrosis is closely associated with the growth in DN [8]. The clinical manifestations and symptoms of kidney fibrosis include increased proteinuria, decreased glomerular filtration rate, hypertension, edema, anemia, and uremic syndrome [9] (Figure 1). The detrimental effects of kidney fibrosis primarily lie in its disruption of normal kidney structure and function, leading to the gradual loss of physiological functions such as glomerular filtration, tubular function impairment, and vascular structural changes [10]. Given the significance of fibrosis progression, investigating the interplay of pathways and targets offers important insights for therapeutic interventions aimed at delaying fibrosis.

Current research should not only explore fibrosis mechanisms at the microscopic level but also identify new materials and methods from a macroscopic perspective to provide potential therapeutic directions for DN treatment. There is an urgent need to find new, highly effective drug candidates with few side effects to improve DN. Natural polyphenols, widely present in fruits, vegetables, spices, and herbs, have shown significant potential in alleviating the pathological progression of DN by improving podocyte detachment and apoptosis, inhibiting kidney tubulointerstitial fibrosis, controlling cell proliferation, reducing excessive extracellular matrix production by mesangial cells, and regulating macrophage infiltration and phenotypic changes [11]. This article briefly explores the molecular mechanisms, kidney cell types, and signaling pathways in DN, and also reviews the structures and pharmacological activities of natural polyphenols, as well as polyphenols targeting the antifibrotic amelioration of DN, which provide a potential research strategy for exploring the amelioration of DN using natural polyphenols. Furthermore, in addition to being readily available, polyphenols combine the key characteristics of natural products: low toxicity, high safety, a wide range of targets and modes of action, all of which are important guarantees for further research. They provide potential therapeutic drugs and therapeutic strategies for polyphenols to be developed as a preventive treatment for diabetic nephropathy.

## 2. Kidney Fibrosis in Diabetes

### 2.1. Signaling Pathways Related to Kidney Fibrosis

The progression of fibrosis in diabetic nephropathy involves multiple complex signaling pathways, which play crucial roles in regulating fibrosis. A comprehensive understanding of the mechanisms of these signaling pathways not only makes it possible to uncover the pathological process of fibrosis, but also provides an important basis for the development of new therapeutic strategies. The most important signaling pathways that play a key role in renal fibrosis and their regulatory mechanisms are now briefly discussed.

#### 2.1.1. TGF-β/SMAD

**Figure 2:**
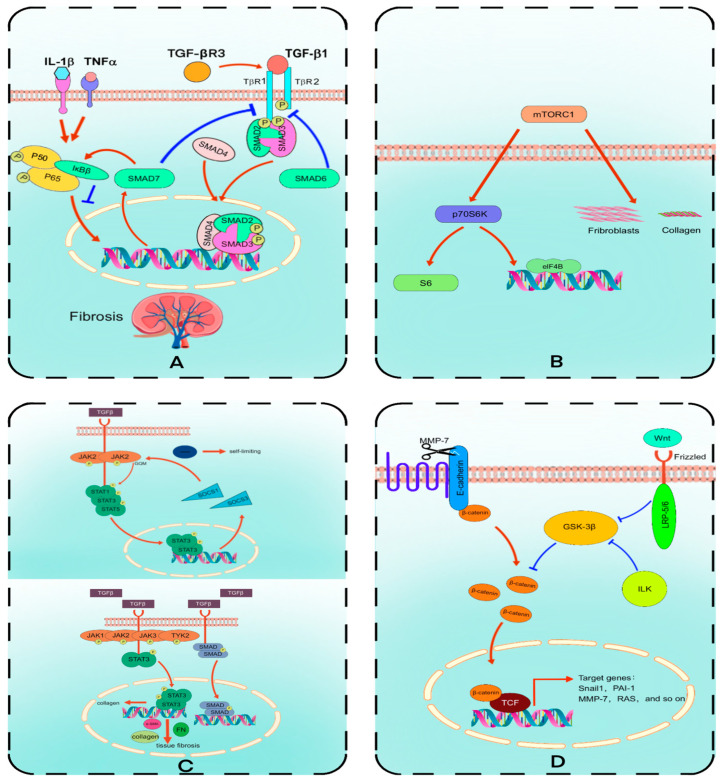
Key signaling pathways involved in kidney fibrosis. (**A**) TGF-β/SMAD signaling pathway. TGF-β activates SMAD2/3 through binding to its receptors, promoting the expression of fibrosis-related genes. (**B**) mTORC1/p70S6K signaling pathway. mTORC1 activates p70S6K, which subsequently promotes protein synthesis and the production of fibrosis-related proteins. (**C**) JAK/STAT/SOCS signaling pathway. JAK kinases activate STAT proteins, promoting the transcription of fibrosis-related genes, while SOCS proteins regulate this pathway via negative feedback. (**D**) Wnt/β-catenin signaling pathway. Wnt signaling stabilizes β-catenin by inhibiting GSK-3β, ultimately promoting the expression of fibrosis-related genes. Arrows in the figure indicate activation or promotion, while markers with lines through them represent inhibition or blockage.

In addition to the main components of fibrosis, such as collagen, fibronectin, and other extracellular matrix (ECM) proteins [12,13], several important regulatory proteins are involved in kidney fibrosis. Transforming growth factor-β (TGF-β), a multifunctional cytokine, plays a critical role in kidney fibrosis by binding to its receptors and initiating a cascade of intracellular signaling events. This regulates cell growth, differentiation, and fibrosis [14] and is central to the TGF-β/SMAD signaling pathway. When TGF-β binds to its receptors, TGF-βR1 and TGF-βR2, TGF-βR2 phosphorylates and activates TGF-βR1, initiating the signaling cascade [15]. The activated TGF-βR1 then phosphorylates R-SMAD proteins (primarily SMAD2 and SMAD3), allowing them to translocate from the cytoplasm to the nucleus [16]. In the nucleus, phosphorylated SMAD2/3 form transcriptional complexes with co-SMAD (SMAD4), which bind to the promoter regions of target genes to regulate the expression of fibrosis-related genes [17] (Figure 2A).

The TGF-β/SMAD signaling pathway also involves other key regulatory proteins. For example, TGF-βR3 (beta glycan), an accessory receptor lacking tyrosine kinase activity, enhances the binding of TGF-β to TGF-βR2, thereby promoting signal transduction. Additionally, I-SMADs (SMAD6 and SMAD7) negatively regulate TGF-β signaling by inhibiting the activation and nuclear translocation of R-SMADs. The SMAD anchor for receptor activation (SARA) functions as an anchoring protein for SMAD2/3 in the cytoplasm, helping SMAD2/3 localize to the TGF-β receptor complex to facilitate their phosphorylation and activation.

Numerous studies and clinical data have demonstrated that inhibiting the TGF-β/SMAD signaling pathway can effectively slow down or reverse kidney fibrosis. For example, TGF-β neutralizing antibodies or small molecule inhibitors can block the binding of TGF-β to its receptors, thereby reducing the expression of fibrosis-related genes [18]. Research shows that using TGF-β neutralizing antibodies or small molecule inhibitors such as galunisertib effectively blocks the binding of TGF-β to its receptors, reducing SMAD2/3 phosphorylation levels by approximately 70%, and decreasing the expression of fibrosis genes such as collagen and fibronectin. Inhibitors targeting SMAD3 have also demonstrated good anti-fibrotic effects, preventing SMAD3 phosphorylation and nuclear translocation. Studies have found that using a specific SMAD3 cell-penetrating peptide (CPP) reduced SMAD3 nuclear translocation by about 80%, leading to a nearly 60% decrease in the expression of fibrosis markers [19]. Clinically, a phase II trial of LY2382770, a monoclonal antibody that specifically neutralizes TGF-β1, was conducted in patients with moderate to severe diabetic nephropathy.

#### 2.1.2. mTORC1/p70S6K

mTORC1 (mechanistic target of rapamycin complex 1) is a key regulator of cell growth, proliferation, and metabolism. By integrating various external signals such as nutrient availability, energy status, and growth factor levels, mTORC1 regulates numerous cellular functions [20].

The mTORC1/p70S6K signaling pathway promotes kidney fibrosis by regulating protein synthesis. Upon activation of p70S6K by mTORC1, p70S6K further phosphorylates and activates translation initiation factors such as eIF4B and ribosomal protein S6, which play critical roles in protein translation and ribosome biogenesis (Figure 2B). Through this mechanism, the mTORC1/p70S6K signaling pathway significantly enhances the synthesis of fibrosis-related proteins such as collagen and fibronectin [21].

An experimental study by Wang demonstrated that in a model of kidney fibrosis, the phosphorylation level of the downstream effector protein p70S6K in the mTORC1 pathway was significantly elevated, approximately 2.5 times higher than that of the control group. This finding highlights the overactivation of the mTORC1/p70S6K pathway as a critical feature of kidney fibrosis [22]. Furthermore, inhibiting the mTORC1/p70S6K pathway can effectively slow or reverse kidney fibrosis. For instance, rapamycin, a specific mTORC1 inhibitor, has been shown to significantly reduce the synthesis of fibrosis-related proteins and the proliferation of fibroblasts, thereby exerting anti-fibrotic effects [23]. Experiments conducted on a unilateral ureteral obstruction (UUO) mouse model and primary fibroblasts found that a p70S6K inhibitor, by preventing p70S6K phosphorylation, significantly reduced the expression of fibrosis-related genes, thus demonstrating anti-fibrotic effects [24].

#### 2.1.3. JAK/STAT/SOCS

The activation of the JAK/STAT/SOCS signaling pathway begins with the binding of cytokines to their respective receptors, which subsequently activates receptor-associated Janus kinases (JAKs). The JAK family includes JAK1, JAK2, JAK3, and TYK2 [25]. Once activated, JAKs phosphorylate specific receptor sites, providing docking points for signal transducers and activators of transcription (STATs) [26].

Upon phosphorylation by JAKs, STAT proteins dimerize and translocate to the nucleus, where they bind to specific DNA sequences, initiating the transcription of fibrosis-related genes [27]. These genes include type I and type III collagen, fibronectin, and α-smooth muscle actin (α-SMA), whose overexpression is characteristic of kidney fibrosis [28] (Figure 2C). Additionally, the persistent activation of STAT3 is a critical feature of fibrosis, as it drives the activation and excessive proliferation of fibroblasts, accelerating the fibrosis process [29].

Meanwhile, suppressors of cytokine signaling (SOCS) proteins play a key role in regulating the JAK/STAT pathway. The SOCS family, which includes SOCS1 to SOCS7 and cytokine-induced SRC homology (CIS) proteins, inhibits JAK kinase activity through negative feedback mechanisms, thereby controlling the intensity and duration of signaling [30]. In kidney fibrosis, SOCS1 and SOCS3 are particularly important, as they inhibit JAK/STAT signaling by binding to JAK and promoting its degradation [31].

In an experiment using an ob/ob mouse model, the authors applied a peptide mimetic of SOCS1 (MiS1) and showed that treatment with MiS1 significantly reduced the levels of phosphorylated STAT3 and the expression of fibrosis markers (such as collagen and fibronectin) also significantly decreased (by more than 50%). The results suggest that through the action of SOCS1, inhibiting JAK/STAT signaling can effectively reduce the progression of kidney fibrosis [32].

#### 2.1.4. Wnt/β-Catenin

Studies have reported that activation of Wnt contributes to the repair and regeneration of damaged kidney tubules. However, the long-term activation of Wnt exacerbates fibrotic nephropathy [33]. Wnt signaling activates downstream pathways by binding to frizzled receptors and low-density lipoprotein receptor-related proteins (LRP5/6) on the cell membrane, inhibiting glycogen synthase kinase-3β (GSK-3β) and preventing β-catenin degradation [34]. During this process, integrin-linked kinase (ILK) also plays a role in regulating GSK-3β, further stabilizing β-catenin. As β-catenin accumulates in the cytoplasm, it is released and translocates to the nucleus [35] (Figure 2D).

In the nucleus, β-catenin binds to T-cell factor (TCF), initiating the transcription of a range of target genes, including Snail1, PAI-1, MMP-7, and RAS, which are closely related to cell proliferation, differentiation, and ECM production [36]. Additionally, the degradation of E-cadherin leads to the release of more β-catenin, further enhancing Wnt signaling. This pathway promotes fibrosis by activating and proliferating fibroblasts, accelerating the excessive production of ECM components such as collagen and fibronectin [37].

Experimental studies have shown that the Wnt/β-catenin pathway is significantly activated in kidney fibrosis. For example, in the UUO mouse model, activation of the Wnt/β-catenin pathway led to a threefold increase in the expression of fibrosis-related genes compared to the control group. Inhibition of the Wnt/β-catenin pathway can significantly slow or reverse kidney fibrosis. IWP-2, a Wnt signaling inhibitor, has been shown to significantly reduce the synthesis of fibrosis-related proteins and the proliferation of fibroblasts, thereby exerting anti-fibrotic effects [38]. Furthermore, studies have found that the expression of key proteins in the Wnt/β-catenin pathway, such as β-catenin and TCF/LEF, is significantly increased in the kidney tissues of DN patients and positively correlates with the degree of fibrosis.

### 2.2. Potential Mechanisms of Kidney Fibrosis

The pathological mechanisms of kidney fibrosis involve complex interactions between various cell types and signaling pathways. TGF-β is a central mediator in the fibrosis process, playing a critical role by activating fibroblasts and promoting the synthesis of extracellular matrix (ECM) components. Additionally, oxidative stress and inflammatory responses are important drivers of fibrosis, exacerbating kidney injury by promoting apoptosis, ECM deposition, and the release of inflammatory cytokines. A brief overview of fibrosis and the underlying mechanisms is provided below.

#### 2.2.1. Fibrosis and Autophagy

**Figure 3:**
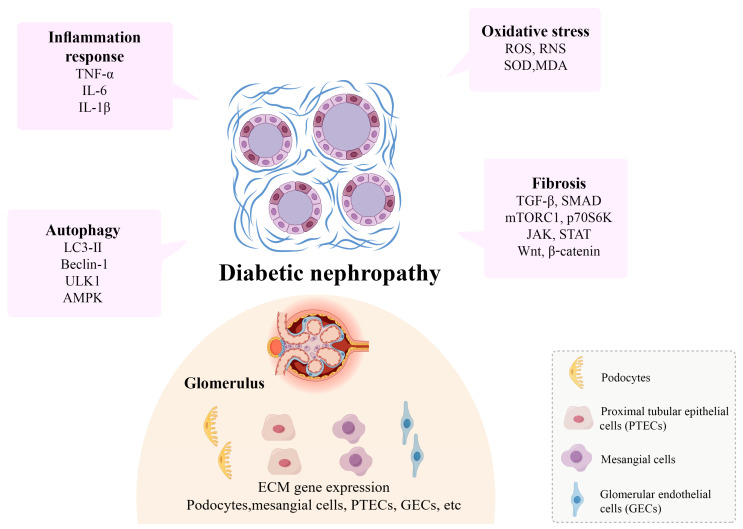
Major pathogenic mechanisms and influencing factors of kidney fibrosis. (Part of the picture is drawn by Figdraw (http://www.figdraw.com, access on 13 November 2024)).

Autophagic degradation mechanisms play a critical role in the pathogenesis of kidney fibrosis. Autophagy maintains cellular homeostasis and function by clearing damaged organelles and proteins, which is crucial in preventing fibrosis [39]. In various models of kidney fibrosis, autophagy activity is significantly decreased, particularly when there is excessive accumulation of ECM components in fibroblasts, with autophagy inhibition being more pronounced. For example, in the UUO model, the expression of autophagy-related proteins such as LC3-II and Beclin-1 is markedly reduced, indicating suppressed autophagic activity [40] (Figure 3).

Multiple signaling pathways play key roles in regulating the relationship between autophagy and kidney fibrosis. mTORC1 is an important negative regulator of autophagy. By inhibiting the activity of the ULK1 complex, mTORC1 suppresses the initiation of autophagy [41]. In kidney fibrosis, overactivation of mTORC1 not only inhibits autophagy but also promotes fibroblast proliferation and ECM synthesis, thereby exacerbating fibrosis [42]. For instance, studies have shown that the mTORC1 inhibitor rapamycin significantly alleviates fibrosis by activating autophagy [43].

AMPK (AMP-activated protein kinase) is another key regulator of autophagy. By inhibiting mTORC1 and directly activating ULK1, AMPK promotes the induction of autophagy. In kidney fibrosis, AMPK activity is typically suppressed, leading to decreased autophagy and aggravated fibrosis [44]. Activating AMPK can restore autophagy, reduce the accumulation of fibrosis-related proteins, and thus exert anti-fibrotic effects [45].

The TGF-β signaling pathway also plays a critical role in kidney fibrosis [46]. TGF-β inhibits autophagy by upregulating mTORC1 activity and downregulating the expression of autophagy-related genes. TGF-β not only directly inhibits autophagy but also promotes fibroblast activation and ECM synthesis by activating downstream SMAD signaling, thereby aggravating fibrosis [42].

In addition to these signaling pathways, the FoxO (Forkhead Box O) family of transcription factors plays a significant role in regulating both autophagy and fibrosis. FoxO3 is a key regulator of autophagy, promoting its occurrence by upregulating autophagy-related genes such as LC3 and Beclin-1. In kidney fibrosis, FoxO3 activity is typically suppressed, leading to decreased autophagy and worsening fibrosis. Activating FoxO3 can significantly increase autophagic activity and reduce fibrosis [47].

#### 2.2.2. Fibrosis and Oxidative Stress

Oxidative stress plays a crucial role in the pathogenesis of kidney fibrosis. It arises from an imbalance between the excessive production of reactive oxygen species (ROS) and reactive nitrogen species (RNS) or the dysregulation of antioxidant defense mechanisms [48]. In kidney fibrosis, ROS promote the fibrotic process through various mechanisms. First, ROS can directly damage the DNA, proteins, and lipids of kidney cells, inducing apoptosis and necrosis, which in turn lead to the destruction of kidney structures [49]. Additionally, ROS activate several signaling pathways, such as TGF-β/SMAD, MAPK, and NF-κB, further promoting the expression of fibrosis-related genes [50] (Figure 3).

The TGF-β/SMAD signaling pathway plays a central role in ROS-mediated kidney fibrosis. ROS enhance the expression and activation of TGF-β, amplifying TGF-β/SMAD signaling, thereby upregulating fibrosis-related genes such as collagen and fibronectin [51]. Specifically, after binding to its receptor, TGF-β activates SMAD2/3, which translocate to the nucleus, bind to the promoter regions of target genes, and regulate the transcription of fibrosis-related genes [52]. Additionally, ROS regulate non-SMAD pathways, such as MAPK (mitogen-activated protein kinase), which also play an important role in kidney fibrosis [53]. The MAPK family, including ERK (extracellular regulated protein kinases), JNK (c-Jun N-terminal kinase), and p38, promotes the expression of fibrosis-related genes through the phosphorylation of specific transcription factors [54].

Moreover, the Nrf2 (nuclear factor erythroid 2-related factor 2) signaling pathway, an essential component of the cellular antioxidant defense mechanism, also plays a protective role in kidney fibrosis [55]. Nrf2 regulates the expression of antioxidant genes, reducing ROS accumulation and thereby mitigating oxidative stress-induced cellular damage. In kidney fibrosis, Nrf2 activity is typically suppressed, leading to decreased antioxidant capacity and elevated ROS levels. Activating the Nrf2 signaling pathway can significantly reduce the expression of fibrosis-related genes and alleviate fibrosis [56] (Figure 3).

#### 2.2.3. Fibrosis and Inflammation

Kidney fibrosis is a key pathological feature in the progression of chronic kidney disease (CKD), with inflammation playing a critical role in this process. Inflammatory responses promote the onset and progression of kidney fibrosis through various mechanisms [57]. Inflammatory cells such as macrophages, neutrophils, and T cells infiltrate the kidneys, releasing pro-inflammatory factors such as TNF-α, IL-6, and IL-1β. These factors, by activating their respective receptors and signaling pathways, further exacerbate inflammation and fibrosis [58]. The Toll-like receptor 4 (TLR4) and nuclear factor κB (NF-κB) signaling pathways play central roles in the interplay between kidney inflammation and fibrosis. TLR4 recognizes pathogen-associated molecular patterns (PAMPs) and damage-associated molecular patterns (DAMPs), activating downstream MyD88-dependent and MyD88-independent signaling pathways, ultimately leading to NF-κB activation [59] (Figure 3).

NF-κB is a key transcription factor that promotes kidney fibrosis by regulating the expression of various inflammatory and fibrosis-related genes [60]. Studies have shown that inhibiting the TLR4/NF-κB signaling pathway significantly reduces kidney interstitial fibrosis in diabetic nephropathy. One specific mechanism includes the upregulation of S100A8/A9 proteins in the TLR4/NF-κB pathway, which exacerbates fibrosis by enhancing epithelial–mesenchymal transition (EMT) and ECM deposition. Additionally, the NLRP3 inflammasome plays an important role in kidney fibrosis. NLRP3 detects intracellular danger signals, activates Caspase-1, and promotes the maturation and secretion of IL-1β and IL-18, thereby aggravating inflammation and fibrosis [61]. Studies have demonstrated that apocynin can alleviate kidney fibrosis in diabetic nephropathy by inhibiting the NLRP3/XIAP signaling pathway [50].

The relationship between the NLRP3 inflammasome and fibrosis is particularly close. Specifically, NLRP3 activation promotes fibroblast activation and ECM synthesis by upregulating the expression of TGF-β and other fibrosis-related factors [62]. Inhibiting the NLRP3 inflammasome can significantly reduce the expression of fibrosis-related genes and alleviate kidney fibrosis. Moreover, XIAP (X-linked inhibitor of apoptosis protein) plays a key role in NLRP3 inflammasome-mediated fibrosis. XIAP protects cells from apoptosis by inhibiting Caspase-3 and Caspase-7 while promoting the fibrosis process [63].

### 2.3. Fibrotic Niche in Kidney Fibrosis

Kidney homeostasis is a critical factor in maintaining overall health, and the development of fibrosis can severely disrupt this balance. Kidney fibrosis not only affects the function of the glomeruli and tubules but also involves complex interactions between various cell types. These cell types each play important roles in the fibrotic process, including podocytes, proximal tubular epithelial cells (PTECs), mesangial cells, and glomerular endothelial cells (GECs). Although the major cell types of the fibrotic ecological niche have been identified, the functional heterogeneity and interactions between the cell lines remain to be clarified.

#### 2.3.1. Podocytes

Podocytes play an essential role in the glomerular filtration barrier. These specialized cells, located on the glomerular basement membrane (GBM), possess complex projections known as foot processes, which adhere tightly to the GBM, forming filtration slits. These slits allow water and small molecules to pass through while preventing the passage of large molecules and cells. In kidney fibrosis, podocyte injury and dysfunction are critical events that lead to the disruption of the filtration barrier and the onset of proteinuria [64] (Figure 3).

Podocyte injury is often accompanied by cytoskeletal remodeling and apoptosis, processes triggered by various mechanisms, including hyperglycemia, oxidative stress, and inflammatory factors. In hyperglycemic conditions, the levels of AGEs in podocytes increase. These AGEs bind to their receptor RAGE, activating downstream signaling pathways such as TGF-β/SMAD and NF-κB, leading to cytoskeletal remodeling and podocyte detachment [65]. Furthermore, the accumulation of ROS in podocytes is another significant mechanism of podocyte injury. ROS induce apoptosis and functional loss by oxidizing proteins and lipids [66].

Podocyte injury and loss also result in the release of numerous pro-fibrotic factors such as connective tissue growth factor (CTGF) and vascular endothelial growth factor (VEGF), which activate other cells in the glomerulus, including mesangial and endothelial cells, further promoting fibrosis [67]. Studies have shown that preserving podocyte function or preventing podocyte injury can significantly slow the progression of kidney fibrosis. For example, using podocyte-specific TGF-β inhibitors reduces the excessive deposition of ECM proteins and improves glomerular filtration function [68]. As a key component of the glomerular filtration barrier, podocytes are central regulatory nodes in various fibrotic signaling pathways.

#### 2.3.2. Proximal Tubular Epithelial Cells

Proximal tubular epithelial cells (PTECs) play a crucial role in the filtration and reabsorption processes of the kidney. These cells maintain fluid and electrolyte balance and acid-base homeostasis by reabsorbing water, electrolytes, and nutrients. The dense microvilli on PTECs dramatically increase their surface area, enhancing reabsorption efficiency. During kidney fibrosis, the dysfunction and structural disruption of PTECs become key factors in the progression of fibrosis [69] (Figure 3).

Under pathological conditions such as hyperglycemia and inflammation, PTECs are significantly affected. Hyperglycemia damages PTECs through various mechanisms, including oxidative stress and the accumulation of AGEs. Oxidative stress leads to the overproduction of ROS, which damage the DNA, proteins, and lipids of PTECs, inducing apoptosis and fibrosis [70]. At the same time, AGEs bind to their receptor RAGE, activating downstream signaling pathways such as MAPK and NF-κB, further exacerbating inflammation and fibrosis.

In hyperglycemic conditions, the expression of TGF-β is significantly increased. After binding to its receptor, TGF-β activates SMAD2/3, which translocate to the nucleus and regulate the expression of fibrosis-related genes such as collagen and fibronectin. The overexpression of these fibrosis genes leads to ECM accumulation around the tubules, further aggravating tubular structural damage and functional loss [71,72].

Simultaneously, the Wnt/β-catenin signaling pathway also plays a vital role in the fibrosis process of PTECs. Under hyperglycemic conditions, the expression of Wnt signaling molecules is significantly increased. These molecules bind to their frizzled receptor, activating β-catenin, which translocates to the nucleus to promote the expression of fibrosis-related genes. Inhibiting the Wnt/β-catenin signaling pathway can significantly reduce kidney fibrosis [73].

During inflammatory responses, hyperglycemia and oxidative stress induce PTECs to release large amounts of pro-inflammatory factors such as TNF-α and IL-6. These factors, through the activation of the NF-κB signaling pathway, further exacerbate inflammation and fibrosis. Once NF-κB is activated, it transcribes numerous pro-inflammatory and pro-fibrotic genes, leading to tissue damage and the progression of fibrosis [74].

Under hyperglycemic conditions, PTECs upregulate ET-1, promoting vasoconstriction and kidney ischemia, exacerbating kidney injury and fibrosis. ET-1, upon binding to its receptor, activates several downstream signaling pathways, including RhoA/ROCK and PI3K/AKT, promoting the expression of fibrosis-related genes and the synthesis of ECM [75]. As a critical component of tubular reabsorption function, PTECs are also central regulatory nodes in multiple fibrotic signaling pathways.

#### 2.3.3. Mesangial Cells

Mesangial cells, located in the central region of the glomerulus, perform various functions, including supporting the glomerular structure, regulating blood flow, and participating in immune responses. They maintain the normal metabolism and balance of the glomerular matrix by synthesizing ECM and regulating the activity of matrix metalloproteinases (MMPs). During kidney fibrosis, abnormal activation and proliferation of mesangial cells are key events driven by pathological factors such as hyperglycemia, inflammation, and oxidative stress [76] (Figure 3).

Under hyperglycemic conditions, mesangial cells undergo pathological changes through multiple signaling pathways. Hyperglycemia-induced oxidative stress increases ROS production, leading to cell damage and inflammatory responses [77]. ROS not only directly damage mesangial cells but also promote their proliferation and excessive ECM deposition by activating the TGF-β signaling pathway. TGF-β binds to its receptors, activating downstream SMAD2/3, which translocate to the nucleus and regulate the expression of fibrosis-related genes, such as collagen and fibronectin [78].

In addition to the TGF-β signaling pathway, AGEs are also important drivers of pathological changes in mesangial cells. AGEs bind to their receptor RAGE, activating pathways such as MAPK and NF-κB, which exacerbate inflammation and fibrosis. The MAPK signaling pathway regulates cell proliferation and ECM synthesis by phosphorylating specific transcription factors, leading to abnormal proliferation and fibrosis in mesangial cells [79].

The NF-κB signaling pathway plays a crucial role in the inflammatory and fibrotic responses of mesangial cells. Hyperglycemia and oxidative stress induce NF-κB activation, which translocates to the nucleus and initiates the transcription of pro-inflammatory and pro-fibrotic genes, such as TNF-α, IL-6, and MCP-1. These inflammatory factors further aggravate mesangial cell inflammation and excessive ECM accumulation, driving the progression of fibrosis [80].

Furthermore, mesangial cells influence the behavior of surrounding cells during fibrosis by secreting various growth factors and cytokines. For example, CTGF secreted by mesangial cells promotes proliferation and fibrosis through autocrine and paracrine mechanisms. Additionally, VEGF secreted by mesangial cells, while promoting angiogenesis, also participates in the fibrotic process [81]. Mesangial cells are not only a major component of the glomerular matrix but also regulate fibrosis through multiple signaling pathways and secreted factors.

#### 2.3.4. Glomerular Endothelial Cells

Glomerular endothelial cells (GECs) play an essential role in glomerular function, forming the endothelial layer of the glomerular filtration barrier that regulates the exchange of substances between the blood and filtrate [82]. GECs possess highly specialized fenestrations that allow water and small molecules to pass freely while preventing the passage of large proteins and cells. In the pathology of kidney fibrosis, GEC injury and dysfunction are considered critical events, driven by factors such as hyperglycemia, inflammation, and oxidative stress [83] (Figure 3).

Under hyperglycemic conditions, GECs undergo significant pathological changes. Hyperglycemia-induced oxidative stress increases ROS production, leading to GEC damage and inflammatory responses. ROS not only directly damage endothelial cells but also promote endothelial–mesenchymal transition (EndMT), a process crucial in kidney fibrosis, by activating the TGF-β signaling pathway. TGF-β binds to its receptors, activating downstream SMAD2/3, which translocate to the nucleus and regulate the expression of fibrosis-related genes such as collagen and fibronectin [84].

AGEs, which accumulate in the diabetic environment, also drive fibrosis by binding to their receptor RAGE and activating a series of downstream signaling pathways, including MAPK and NF-κB, further exacerbating inflammation and fibrosis. The MAPK signaling pathway regulates cell proliferation and ECM synthesis by phosphorylating specific transcription factors, leading to abnormal proliferation and fibrosis in GECs [85].

Inflammatory responses also play a key role in GEC injury and fibrosis. Hyperglycemia and oxidative stress induce NF-κB activation, which translocates to the nucleus and initiates the transcription of pro-inflammatory and pro-fibrotic genes such as TNF-α, IL-6, and MCP-1. These inflammatory factors further exacerbate endothelial cell inflammation and excessive ECM accumulation, driving the progression of fibrosis [86].

Endothelial cell injury not only affects their function but also influences surrounding cells through the release of cytokines and growth factors. For example, VEGF secreted by GECs promotes angiogenesis and participates in the fibrotic process. VEGF binds to its receptor and activates the PI3K/AKT signaling pathway, promoting the expression of fibrosis-related genes and ECM synthesis [87].

The dysfunction of GECs also exacerbates kidney fibrosis through the activation of the renin–angiotensin system (RAS). Under hyperglycemic conditions, the levels of angiotensin II (Ang II) are significantly increased. Ang II binds to its receptor, activating several downstream signaling pathways such as JAK/STAT and RhoA/ROCK, promoting endothelial inflammation and fibrosis. Inhibiting RAS can significantly reduce GEC injury and slow the progression of kidney fibrosis [88]. Glomerular endothelial cells are not only integral to glomerular filtration function but also serve as key regulatory nodes in various fibrotic signaling pathways. Currently, potential targets and therapeutic strategies at the molecular level have been identified for these pathways and mechanisms. However, clinical treatment of DN should go beyond these targets and be combined with pharmacotherapy to achieve optimal outcomes. Presently, combination therapy is commonly employed in DN treatment, and natural polyphenols provide a broad range of material sources for this purpose.

## 3. Polyphenols and Their Applications

In general, the potential for therapeutic agents lies in targeting these key mechanisms. Although the current routine use of drugs such as renin angiotensin aldosterone system (RAAS) blockers, SGLT2 inhibitors, and GLP-1 receptor agonists can delay the progression of chronic kidney disease (CKD) to varying degrees [57]. SGLT-2i reduces glucose levels in the circulation by decreasing glucose reabsorption in urine, primarily through inhibition of renal tubular SGLT2. The SGLT-2i currently available are dapagliflozin, engliflozin, canagliflozin and ertugliflozin. It has been found that SGLT2i monotherapy can reduce HbA1c by 0.5~1.2% and improve end-stage renal disease at the same time. However, it is associated with reproductive system infections and adverse reactions, which are related to blood volume insufficiency [89]. Treatments targeting a single pathological phenotype may lead to unpredictable events in the course of complications. This is why traditional Chinese medicine (TCM) therapeutic strategies with multiple components, multiple targets and multiple pathways have emerged. Multiple components in natural products: polyphenols, alkaloids, lignans, steroids, protein peptides and sugars provide a wide range of material sources. The chemical structure of polyphenols and the pharmacological properties of improved DN are now being studied.

### 3.1. Polyphenol Chemistry

In the pathological progression of kidney fibrosis, various signaling pathways interact, leading to irreversible kidney damage. Given the importance of these mechanisms, the search for natural compounds capable of modulating these pathways has become a key focus of research [90]. Polyphenolic compounds are widely found in the plant kingdom and are a class of chemicals characterized by having at least one phenolic group (-OH) attached to an aromatic ring. They have garnered significant attention in scientific research due to their rich biological activities, particularly their potent antioxidant and anti-inflammatory properties [91]. The structural diversity of polyphenols leads to their broad classification, with each group of compounds possessing specific biological functions and chemical properties that demonstrate potential utility in the prevention and treatment of various diseases [92].

Flavonoids: flavonoids represent the largest subclass of polyphenols, characterized by a basic C6-C3-C6 structure, which includes two aromatic rings (A and B rings) connected by a three-carbon chain (C ring). Based on the configuration of the C ring and the position of hydroxyl groups, flavonoids are further divided into several subclasses, including the following:

Flavonols and flavones are widely present in fruits and vegetables and have notable health benefits. According to a study by Panche, these two types of flavonoids exhibit excellent antioxidant and anticancer effects [93]. These effects are partly attributed to their unique chemical structure—two aromatic rings (A and B rings) connected by a three-carbon C ring. This basic C6-C3-C6 structure is crucial to their bioactivity. The primary difference between flavonols and flavones lies in the substitution patterns on the B ring. Flavonols typically have at least one hydroxyl group on the B ring, while flavones may lack this feature.

Anthocyanins are the primary pigments responsible for the coloration in many plants and play a core role in various biological functions. These compounds feature an open C ring containing multiple hydroxyl groups, such as epicatechin, a major component of green tea [94]. These hydroxyl groups not only give anthocyanins their vibrant colors but also contribute to their diverse biological activities, such as antioxidant and anti-inflammatory effects.

Isoflavones are a specialized class of flavonoids predominantly found in legumes. Their distinctive feature lies in their C ring, which contains a non-aromatic carbon structure, unlike other flavonoids. This unique configuration gives isoflavones biological activity similar to hormones, particularly estrogen. As a result, isoflavones are often studied for their ability to modulate hormonal activity, especially in the context of phytoestrogen research [95].

Non-flavonoids: phenolic acids typically consist of a single benzene ring with one or more hydroxyl groups, which confer antioxidant properties. Compounds such as caffeic acid and ferulic acid have demonstrated significant antioxidant and anti-inflammatory activity in cellular and animal models [96].

Lignans are a class of compounds formed by two phenylpropanoid units connected by one or more oxygen bridges, creating a distinctive biphenyl structure. Unlike phenolic acids, lignans contain multiple phenyl rings, equipped with hydroxyl and other functional groups [97].

**Figure 4:**
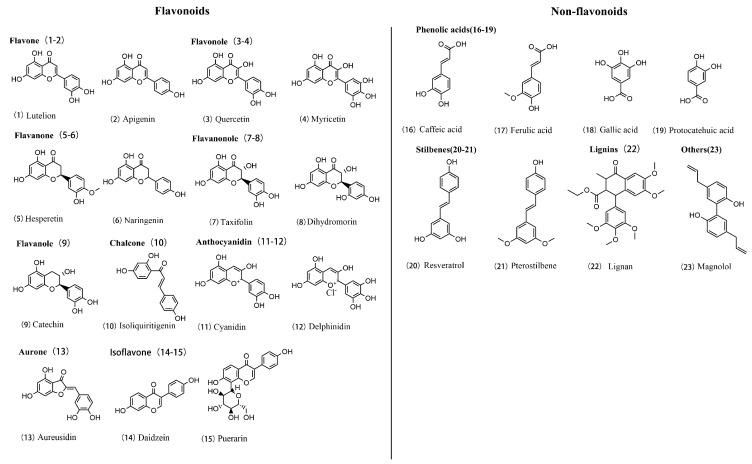
Chemical structures of polyphenolic compounds. Flavonoids on the left, including flavanols, flavanonols, anthocyanins, isoflavones, and others, and non-flavonoids on the right.

Other important polyphenols: tannins are a group of polyphenolic compounds known for their strong ability to bind to proteins and other macromolecules. They are divided into two major types: hydrolysable tannins and condensed tannins. Hydrolysable tannins are based on phenolic acids, such as gallic acid, which are esterified to glucose or other sugars, forming complex networks [98]. These tannins are easily hydrolyzed in water, making them valuable in food preservation and medicine for their antibacterial properties. Condensed tannins, in contrast, are polymers formed from flavanol units, such as catechins, linked by carbon–carbon bonds [99] (Figure 4).

Flavonoid polyphenols, including flavonols, flavones, and anthocyanins, are characterized by the basic C6-C3-C6 skeleton, with their bioactivity primarily attributed to the hydroxyl groups on the B ring, exhibiting strong antioxidant and anti-inflammatory properties. Non-flavonoid polyphenols, such as phenolic acids, tannins, and lignans, have more diverse structures, typically containing one or more phenolic rings without the flavonoid skeleton. These structural differences directly influence the bioactivity of polyphenols, showcasing their wide therapeutic potential in the prevention and treatment of diseases like diabetic nephropathy.

### 3.2. Pharmacological Effects of Polyphenols

Polyphenolic compounds exhibit a variety of biological activities due to their structural characteristics, particularly in regulating inflammation, alleviating oxidative stress, promoting autophagy, and preventing DN (Table 1). Numerous studies have demonstrated that polyphenols can modulate key signaling pathways, such as NF-κB and MAPK, significantly inhibiting the production of pro-inflammatory cytokines, thereby effectively reducing chronic inflammatory responses. This effect is especially pronounced in inflammation-related kidney damage associated with diabetes. According to a study, anthocyanins provide anti-inflammatory and anticancer effects through the hydroxyl groups on their C-ring [100]. These hydroxyl groups effectively scavenge free radicals and alleviate inflammatory responses by inhibiting inflammation-related signaling pathways, which is particularly important in cancer research. In addition to hydroxyl groups, other substituents such as methoxy groups (-OCH₃) and acyl groups (-CO) also play critical roles in enhancing the biological activities of polyphenols [101]. Methoxy groups, often found in compounds like quercetin, increase the compound’s lipid solubility, which improves its membrane permeability and bioavailability. This enhanced bioavailability further supports anti-inflammatory and antioxidant effects, making these compounds more effective in reducing oxidative stress [102]. Acyl groups, on the other hand, contribute to regulating lipid metabolism and decreasing cellular apoptosis, which is particularly beneficial in mitigating fibrosis in diabetic nephropathy [103]. Ional groups collectively enhance the ability of polyphenols to target multiple molecular pathways, providing a comprehensive protective effect against the progression of diabetic nephropathy. Furthermore, research emphasized the health benefits of anthocyanins, particularly their roles in regulating blood glucose and enhancing antioxidant capacity. These studies have shown that anthocyanins improve glucose metabolism and increase cellular resistance to oxidative stress, which is of great significance for the prevention and treatment of diabetes and its complications [104].

Oxidative stress is a critical driving factor in the progression of DN. Due to their remarkable antioxidant properties, polyphenols can effectively scavenge ROS, protecting cells from oxidative stress, thereby playing a key role in the prevention and treatment of DN. In Sahakyan’s 2022 study, tannins were explored in detail for their antioxidant and anti-inflammatory properties [105]. The study found that tannins not only directly eliminate ROS but also chelate metals and upregulate the expression of antioxidant enzymes, helping to reduce ROS levels and inhibit oxidative stress, ultimately protecting the kidneys. Additionally, these compounds reduce the risk of cardiovascular disease through antioxidant and anti-inflammatory mechanisms [106].

Autophagy, a vital mechanism for cellular self-protection and the degradation of damaged organelles, tends to be impaired in DN. Polyphenols, through the regulation of signaling pathways such as mTOR and AMPK, can effectively activate autophagy, reduce cellular damage, and promote the recovery of kidney cell function. In the context of diabetic nephropathy fibrosis, polyphenolic compounds significantly reduce the expression of fibrosis-related proteins by inhibiting the TGF-β/SMAD signaling pathway, thereby delaying or reversing the pathological progression of DN [107]. In a study, kaempferol was shown to activate AMPK and inhibit the mTOR signaling pathway, effectively reducing apoptosis in DN and promoting autophagy, thus improving kidney function. In particular, kaempferol significantly reduced the expression of fibrosis-related proteins by inhibiting the TGF-β/SMAD signaling pathway during diabetic nephropathy fibrosis, thereby delaying or reversing the progression of DN. This study provided strong evidence for the antifibrotic effects of polyphenols in DN, particularly their potential to delay or reverse the progression of DN by regulating autophagic pathways [108].

**Table 1:** Polyphenolic monomers and their bioactivities in the treatment of fibrosis.

Polyphenol	Mechanism	Dosage	Experimental Parameters	Efficacy	Reference
Resveratrol	TGF-β/SMAD, NF-κB	20 mg/kg	Model: bleomycin-induced mouse model duration: 21 days administration: intraperitoneal, daily	anti-inflammatory, antioxidant	[109]
Curcumin	TGF-β/SMAD, NF-κB, PI3K/Akt	100 mg/kg	Model: high-fat diet-induced obese mice duration: 8 weeks administration: oral, daily	anti-inflammatory, anticancer	[110]
Proanthocyanidins	TGF-β/SMAD, MAPK	100 mg/kg	Model: MRI images from various disease groups duration: data collection and analysis phase administration: different levels of grey-level discretization on MRI images	antioxidant, improves cardiovascular health	[111]
EGCG	TGF-β/SMAD, NF-κB	5 mg/kg	Model: male Wistar rats duration: 5 weeks A administration: T treadmill exercise training	anticancer, lowers blood lipids	[112]
Gallic acid	TGF-β/SMAD, AMPK	100 mg/kg	Model: rabbit ear hypertrophic scar model duration: 28 days administration: topical application of gallic acid ointment (varied concentrations)	anti-inflammatory, antibacterial	[113]
Protocatechuic acid	NF-κB, PI3K/Akt	20 mg/kg	Model: review article (no specific experimental model) duration: N/A administration: discusses various antioxidant therapies and mechanisms	anti-inflammatory, antioxidant	[114]
Ginkgolide	Wnt/β-catenin, TGF-β/SMAD	60 mg/kg	Model: elderly patients with heart valve disease duration: surgical procedure and assessment phase administration: evaluated risk and benefit through clinical assessments	improves blood circulation, anti-inflammatory	[115]
Luteolin	NF-κB, PI3K/Akt	50 mg/kg	Model: in vitro and in vivo models duration: varies across studies administration: various concentrations and methods	antiallergic, antioxidant	[116]
Baicalin	TGF-β/SMAD, Wnt/β-catenin	100 mg/kg	Model: review article (no specific model) duration: N/A administration: N/A	antioxidant, antidepressant	[117]
Ginsenoside Rg1	TGF-β/SMAD, NF-κB	20 mg/kg	Model: MRI images from various disease groups duration: data collection and analysis phase administration: different levels of grey-level discretization on MRI images	boosts immune system, anti-fatigue	[118]
Tripterygium glycosides	JAK/STAT, TGF-β/SMAD	50 mg/kg	Model: clinical case study duration: clinical observation administration: not specified	immunosuppressive, anticancer	[119]
Naringin	NF-κB, TGF-β/SMAD	80 mg/kg	Model: rats with DMN-induced liver fibrosis duration: not specified administration: Ganshuang granules in varying dosages	antioxidant, anti-inflammatory	[120]
Hesperidin	PI3K/Akt, NF-κB	100 mg/kg	Model: review article duration: N/A administration: N/A	anti-inflammatory, antiviral, improves microcirculation	[121]
Emodin	TGF-β/SMAD, JAK/STAT	40 mg/kg	Model: not specified duration: not specified Administration: dapansutrile	anti-inflammatory, antibacterial	[122]
Apigenin	NF-κB, TGF-β/SMAD	25 mg/kg	Model: review article (no specific model) duration: N/A administration: N/A	anticancer, anti-anxiety	[123]

### 3.3. The Therapeutic Potential of Polyphenols Targeting Fibrosis in the Treatment of DN

Fibrosis is a critical therapeutic target in the treatment of DN. Natural polyphenols have garnered increasing attention due to their broad biological activity and low toxicity [124]. As compounds found in fruits, vegetables, and other plants, polyphenols have demonstrated significant antioxidant, anti-inflammatory, and antifibrotic effects, particularly showing great potential in the treatment of DN [125].

Polyphenols exert their effects through various mechanisms, with their ability to regulate the TGF-β/SMAD signaling pathway being particularly crucial. Certain polyphenols can inhibit the expression and activity of TGF-β, thereby reducing the phosphorylation of SMAD2/3 and suppressing the expression of fibrosis-related genes. This mechanism effectively reduces ECM accumulation and alleviates the progression of kidney fibrosis [126]. In addition to the TGF-β/SMAD pathway, the mTORC1/p70S6K pathway is another important target of polyphenols. mTORC1 promotes fibroblast proliferation and ECM synthesis through the phosphorylation of its downstream effector protein, p70S6K [127].

The uniqueness of polyphenols lies in their multitarget and multi-mechanism action modes, giving them unparalleled advantages in antifibrotic therapy [128]. Polyphenols directly inhibit the expression of fibrosis-related genes through their effects on various fibrotic signaling pathways, and comprehensively mitigate the progression of kidney fibrosis through antioxidant and anti-inflammatory actions. As natural products, polyphenols possess low toxicity and good tolerability, which is an advantage over many synthetic drugs. This natural diversity and safety make polyphenols highly valuable for long-term therapeutic applications.

Moreover, polyphenols excel in regulating oxidative stress [129]. Excessive production of ROS and RNS under hyperglycemic conditions leads to oxidative stress, directly damaging the DNA, proteins, and lipids of kidney cells, inducing apoptosis and fibrosis. Polyphenols reduce oxidative stress by scavenging reactive species, thereby inhibiting the activation of TGF-β/SMAD, MAPK, and NF-κB pathways [130]. Polyphenols also enhance cellular antioxidant capacity by activating the Nrf2 signaling pathway, reducing fibrosis caused by oxidative stress.

In terms of inflammation regulation, polyphenols demonstrate significant advantages. Studies have shown that polyphenols can significantly inhibit the release of inflammatory cytokines such as TNF-α, IL-6, and IL-1β. By inhibiting the TLR4/NF-κB signaling pathway, they reduce the production of inflammatory mediators and mitigate kidney fibrosis [131]. Additionally, polyphenols can reduce inflammation by inhibiting the activation of the NLRP3 inflammasome, thereby exerting antifibrotic effects. This multitarget, multi-mechanism anti-inflammatory action makes polyphenols ideal candidates for treating DN fibrosis [132].

Due to their diverse biological activities, particularly in antioxidant, anti-inflammatory, and antifibrotic effects, polyphenols are emerging as promising candidate compounds in research for the treatment of diabetic nephropathy. The use of a combination of drugs such as metformin and insulin, which can help to manage problems such as insulin resistance, but also problems such as difficulties in drug delivery or absorption, which are improved by nanomaterials that address the initial challenge of drug delivery [133]. However, the current literature lacks a systematic analysis and evaluation of the coverage and depth of research on different polyphenols in diabetic nephropathy and their academic impact. Therefore, this study conducts a review and quantitative analysis of the relevant literature on polyphenolic compounds in the field of diabetic nephropathy over the past decade, aiming to reveal the research trends and academic impact of various polyphenols in this field, provide data support for future research directions, and explore potential research gaps and breakthrough points.

**Figure 5:**
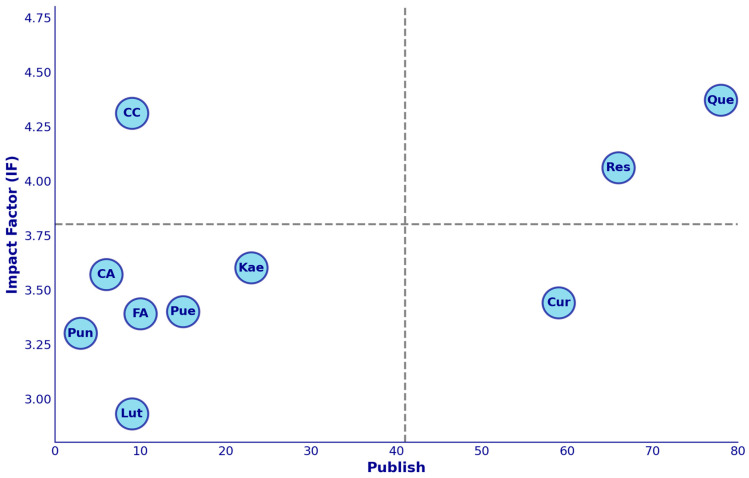
The relationship between the number of publications on polyphenols in the treatment of diabetic nephropathy over the past decade (published) and their average impact factor (IF). The X-axis represents the number of research articles related to each polyphenol, and the Y-axis represents the average impact factor of these studies. The chart is divided into four quadrants, with dashed lines indicating the median number of studies (41) and the median impact factor (3.8). Each bubble represents a specific polyphenol. CA, caffeic acid; Cur, curcumin; CC, cyanidin; FA, ferulic acid; Kae, kaempferol; Lut, luteolin; Pue, pueraria; Pun, punicalagin; Que, quercetin; Res, resveratrol.

As shown in Figure 5, by analyzing the research literature on polyphenols in the treatment of diabetic nephropathy over the past decade, we found that, despite the great therapeutic potential of polyphenols, the existing research remains relatively insufficient. For instance, even the most studied polyphenol, quercetin, has only 78 related publications, while most other polyphenols have even fewer studies. Additionally, although polyphenols like resveratrol and anthocyanins show high impact factors and potential value in the treatment of diabetic nephropathy, the number of studies remains limited. This lack of research may hinder a comprehensive understanding and development of polyphenols for clinical applications. Particularly, some polyphenols, such as anthocyanins and caffeic acid, which show significant research value in specific areas, still require more studies to verify their actual therapeutic effects. Overall, while polyphenols show remarkable potential in the treatment of diabetic nephropathy, more in-depth research is needed to fully explore their clinical value. This research gap reflects the current early stage of the exploration of the potential of polyphenols and their application development in the field of diabetic nephropathy.

DN is a multifactorial complex disease whose pathological progression involves several biological processes, such as fibrosis, oxidative stress, and inflammation. However, the interaction mechanisms between these key processes and their specific roles in different signaling pathways remain largely unexplored. In particular, while studies have suggested that polyphenolic compounds possess potential therapeutic effects in antioxidation, anti-inflammation, and antifibrosis, specific research on their application in the treatment of diabetic nephropathy remains relatively limited.

**Figure 6:**
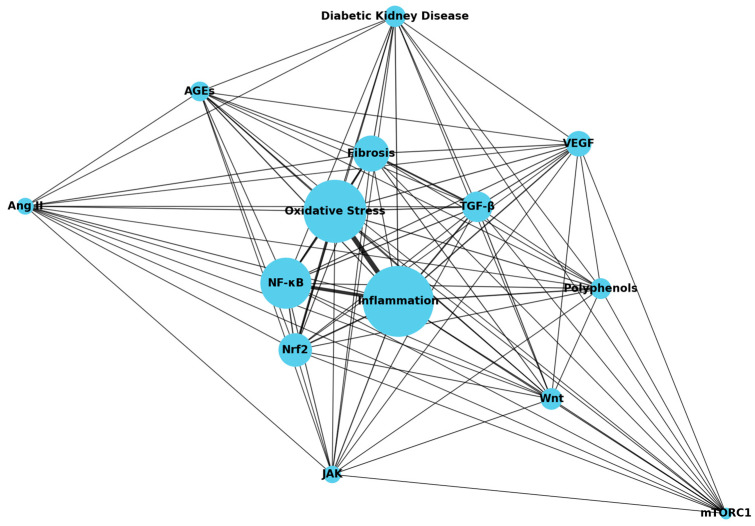
Co-occurrence network of 16 keywords related to DN and its associated biological mechanisms. The size of each node reflects the total occurrence of each keyword in the relevant literature, with larger nodes indicating higher frequency of occurrence. The thickness and intensity of the lines represent the number of times two keywords co-occur in the same literature; the thicker and darker the line, the stronger the association between the two keywords.

As depicted in Figure 6, we constructed a network diagram to systematically reveal the connections between fibrosis, oxidative stress, inflammation, and their related signaling pathways, further exploring the potential role of polyphenolic compounds in the treatment of diabetic nephropathy. The size of each node reflects the frequency of its occurrence in the literature, with larger nodes indicating a higher frequency of occurrence. Notably, NF-κB is an important node, reflecting its central role in the regulation of inflammation and oxidative stress. Line thickness represents the frequency of co-occurrence between keywords; thicker lines indicate stronger associations. These relationships highlight the interconnected nature of key pathological processes in DN and the central role played by specific pathways such as NF-κB and TGF-β. Signaling pathways such as TGF-β, NF-κB, and JAK exhibit strong co-occurrence, supporting the critical role of fibrosis in DN development. NF-κB is a key regulator of inflammation, linked to oxidative stress, where ROS accumulation activates NF-κB, exacerbating inflammation and kidney damage. This positions NF-κB as a central element in the network. In contrast, nodes like Nrf2 are crucial for oxidative stress response and cellular protection. Furthermore, the mTORC1/p70S6K pathway is highlighted for its role in promoting fibroblast proliferation and ECM production, linking metabolism with fibrosis. VEGF, another significant node, is associated with both angiogenesis and fibrotic changes in the diabetic kidney, indicating abnormal vascular processes that contribute to DN. The connections between pathways like VEGF, TGF-β, and JAK illustrate shared regulatory mechanisms impacting fibrosis and inflammation.

The results demonstrate a high degree of interconnection between fibrosis, oxidative stress, and inflammation, indicating that these processes play central roles in the pathogenesis of diabetic nephropathy. Furthermore, multiple signaling pathways closely associated with fibrosis, such as TGF-β, NF-κB, and JAK, exhibit strong co-occurrence, further confirming the critical role of fibrosis in the progression of diabetic nephropathy. This highlights the need for future studies to focus more on the role of polyphenolic compounds in regulating fibrosis, oxidative stress, and inflammation, in order to provide further scientific evidence for therapeutic strategies in diabetic nephropathy.

## 4. Conclusions and Perspectives

This article reviews the effects of fibrosis, autophagy, oxidative stress and inflammation in the fibrotic ecological niche that contribute to the development of DN. As natural compounds with antioxidant and anti-inflammatory properties, polyphenols can ameliorate or reverse cellular damage and pathological changes in DN, and are considered an attractive agent to counter DN by targeting signaling pathways including TGF-β, mTORC1, Wnt, Nrf2, NF-κB, AMPK and SIRT1. However, these studies have some limitations and challenges.

Various studies have used different experimental approaches and models, including in vitro cellular experiments, animal models and a limited number of human clinical trials. While these approaches revealed potential mechanisms of action of the polyphenols, the experimental conditions often differed from the actual physiological environment of the human body, affecting the extrapolation and applicability of the results. A systematic review and meta-analysis, published in 2022, assessed the effects of polyphenols on glycemia, renal, inflammatory and oxidative stress markers in adult DN patients. An analysis of 17 studies found significant reductions in HbA1c levels, proteinuria and malondialdehyde, and an increase in glomerular filtration rate, suggesting a potential renal benefit [134]. Meanwhile, in clinical applications, polyphenols have been found to be challenged by inefficient absorption, rapid metabolism, poor bioavailability, intestinal flora and genetically driven individual differences. Polyphenols can also interact with conventional drugs, affecting metabolism and increasing adverse effects. Despite these limitations, polyphenols still have promising application due to their predominant trend of multi-targeted action, low toxicity and side-effects, and their ability to provide a comprehensive therapeutic strategy for complex diseases such as diabetic nephropathy, which can be tapped into as a target inhibitor to improve the disease by reducing other fibrosis.
